# Pelvic Ring Fractures in Older Adult Patients—Assessing Physician Practice Variation among (Orthopedic) Trauma Surgeons

**DOI:** 10.3390/jcm12196344

**Published:** 2023-10-03

**Authors:** Anna H. M. Mennen, Sharon Oud, Jens A. Halm, Rolf W. Peters, Hanna C. Willems, Daphne Van Embden

**Affiliations:** 1 Department of Surgery, Amsterdam UMC Location University of Amsterdam, Meibergdreef 9, 1105 AZ Amsterdam, The Netherlands; 2 Department of Internal Medicine and Geriatrics, Amsterdam UMC Location University of Amsterdam, Meibergdreef 9, 1105 AZ Amsterdam, The Netherlands

**Keywords:** pelvic ring fracture, pelvic surgery, osteoporotic fracture, diagnostic, treatment, pelvic fragility fracture

## Abstract

Purpose: Pelvic fractures in older adults are a major public health problem and socioeconomic burden. The standard of care has changed over the past years, and there is limited consensus on which patients benefit from surgical fixation. There is currently no nationwide treatment protocol to guide the decision-making process. Therefore, the aim of this survey was to provide more insight into if, when, and why patients with a fragility fracture of the pelvis (FFPs) would be considered for additional imaging and surgical fixation by treating physicians. Methods: An online clinical vignette-based survey of hypothetical scenarios was sent out to all orthopedic and trauma surgeons in the Netherlands. The questionnaire comprised multiple-choice questions and radiographic images. Differences between subgroups were calculated using the X2 test or the Fisher exact test. Results: 169 surgeons responded to the survey, with varying levels of experience and working in different types of hospitals. In a patient with a simple pubic ramus fracture and ASA 2 or ASA 4, 32% and 18% of the respondents would always advise a CT scan for further analysis. In the same patients, 11% and 31% of the respondents would not advise a CT scan, respectively. When presented with three cases of increasing severity of co-morbidity (ASA) and/or increasing age and/or different clinical presentation of an FFP type 3c on a CT scan, an increasing number of respondents would not consider surgical fixation. There was significant variation in practice patterns between the respondents who do not work in a hospital performing pelvic and acetabular (P&A) fracture surgery and those who do work in a P&A referral hospital. Most respondents (77%) refer patients 1–5 times a year to an expert center for surgical fixation. Conclusion: There is currently a wide variety of clinical practices regarding the imaging and management of FFPs, which seems to be influenced by the type of hospital the patients are presented to. A regional or national evidence-based treatment protocol should be implemented to ensure a more uniform approach.

## 1. Introduction

Geriatric trauma is a major public health problem with high absolute numbers and proportions of annual trauma admissions [1,2]. The average annual incidence of pelvic fractures in the Netherlands is 57.9 per 100,000 persons in the older population, and this number is expected to keep rising in the next few decades [3]. Other European countries see a similar trend. In Finland, the total number of pelvic fragility fractures increased by an average of 23% a year between 1970 and 1997, and in Austria, the incidence rate increased by 2.9% between 2010 and 2018 [4,5]. Apart from high morbidity and mortality rates, pelvic fractures in older adult patients also cause a great socioeconomic burden on the healthcare system, with both direct and indirect costs. Patients who suffered a pelvic fragility fracture have a median length of hospital stay of 30 days for women and 39 days for men (overall range 5–170 days), and the 30-day readmission rate of non-operatively treated patients is 12.8%, which is comparable with the readmission rate reported after hip fracture surgery [6,7]. The decreased physical functioning as a result of the sustained injury impacts healthcare costs as well; studies report that 31–43% of patients require temporary or permanent admission to a nursing home after treatment [8,9,10,11].

The diagnosis and management of pelvic fragility fractures (FFPs) have changed over the past few years. Plain pelvic radiographs were the preferred imaging technique to identify pelvic fractures until recently. However, plain radiographic imaging has a sensitivity of only 10.5% in detecting fractures of the posterior pelvic ring in cases of low bone mineral density or superimposed bowel content [12]. Concomitant fractures of the posterior ring found by computed tomography (CT) are reported in up to 54–97% [9,13,14]. Missing fractures of the posterior pelvic ring may have therapeutic consequences, so CT has therefore been recommended as the gold standard for advanced imaging in patients with pelvic pain and inconclusive or negative pelvic radiographs [15]. In the Netherlands, the use of CT to diagnose pelvic fragility fractures is not standard practice, leading to variation in local practice patterns. Unfortunately, (inter)national guidelines for the diagnosis of FFPs are lacking.

The historical standard of care for FFPs has been non-operative treatment. However, experts have increasingly been advocating for the reduction and internal fixation of unstable pelvic fragility fractures to expedite early mobilization and decrease pain complaints to improve postoperative morbidity and functional outcomes [14,16,17]. Furthermore, while pelvic surgery used to be an open procedure with plate fixation spanning over the symphysis or SI joint, current surgical techniques allow for minimally invasive percutaneous fixation with minimal soft tissue disruption. This new technique lowered the threshold for surgical fixation in older adult patients who, in the past, were deemed unfit for surgery. In addition, minimally invasive fixation in these older adult patients allows for faster rehabilitation. However, as there is limited consensus on which patients benefit the most from surgical fixation of their pelvic fragility fracture, the indication for and timing of surgical fixation vary [18]. There is currently no evidence-based treatment protocol for pelvic fragility fractures in the Netherlands to help physicians decide which patients are eligible for surgical fixation or when an expert center should be consulted, which might lead to practice variation and possible undertreatment of vulnerable older patients. This most likely results in a great variety of treatment strategies based on local protocols or the expert opinion of the treating physician.

To assess physician practice variation and local practice patterns, a nationwide clinical vignette-based survey of hypothetical scenarios was performed. We aimed to provide more insight into if, when, and why patients with an FFP would be considered by a responding clinician for additional imaging and surgical fixation.

## 2. Materials and Methods

A nationwide online clinical vignette-based survey was conducted among trauma surgeons and orthopedic surgeons who treat fractures in the Netherlands. The survey was conducted to test the hypothesis that there is currently a physician practice variation in the diagnosis and treatment of pelvic fragility fractures, possibly influenced by the exposure of the respondent to this injury (e.g., hospitals where P&A surgery is performed vs. hospitals that do not perform this type of surgery). The questionnaire was prepared using the online survey tool Qualtrics. All responses were voluntary and anonymized in order to encourage open participation. Surgeons from all general hospitals and university hospitals in the Netherlands were approached through targeted e-mails. In addition, the Dutch Association of Trauma Surgery (NVT) sent the survey to all its members, and the survey was promoted on LinkedIn. Multiple reminders were sent to improve the response rate. There are currently 129 registered orthopedic surgeons who treat fractures in the Netherlands and 370 trauma surgeons. A total of 189 of the 499 registered orthopedic and trauma surgeons responded to the survey. This resulted in an overall response rate of 38%, which is similar to other web-based studies.

The outcome measures of this survey were physician behavior (e.g., would you perform a CT scan? Yes/no) and physician knowledge and attitude (e.g., why perform a CT scan?) towards imaging and treatment of pelvic fragility fractures. To evaluate both of these topics, multiple-choice questions about actual practice patterns and clinical vignettes were included in this survey. First, participants were presented with multiple-choice questions about their general information (e.g., the experience of the respondent, characteristics of the hospital the respondent works in, the level of trauma care, and whether there is exposure to pelvic and acetabular fracture (P&A) surgery), how often they refer patients for surgical fixation, their advice on aftercare (e.g., weight-bearing protocol and follow-up of patients with an FFP), and if there is a local treatment protocol in their hospital.

In addition, participants were presented with five clinical vignettes of older adult patients with a pelvic fragility fracture accompanied by plain radiographic imaging and CT scans (Appendix A, Appendix B and Appendix C). The patients described were all older than 50 years, which was considered the cutoff point to categorize a patient as an older adult with a pelvic fragility fracture. This is based on international guidelines that recommend an assessment of fracture risk in patients over 50 years who have previously sustained a fracture or who have risk factors for osteoporotic fractures [19].

The first two clinical vignettes about imaging described patient A, a 75-year-old woman with ASA 2, who lives independently at home, and patient B, a 77-year-old woman with ASA 4, living in sheltered housing. Both patients showed a ramus superior and inferior fracture on the left side on plain radiographs. These vignettes were presented to determine if and when respondents would perform an additional CT scan or why they would refrain from one.

The three clinical vignettes thereafter described the following patients with FFP type 3c on CT scan: patient C, who is a 75-year-old woman with ASA 2 living independently at home, and patient D, who is a 69-year-old with ASA 3 and COPD Gold 3. Both patients presented at the outpatient clinic after 14 days for difficulty mobilizing indoors using a four-wheel rollator and acetaminophen and NSAIDs for pain management. Patient E is an 81-year-old woman with ASA 3 who was admitted to the nursing ward. She has difficulty turning in bed or making transfers and uses Paracetamol, NSAIDs, and instant Oxycodon pills for pain management. The goal of these three vignettes was to determine if and when respondents deemed the patient eligible for surgical fixation.

Descriptive statistics on the frequencies of baseline characteristics were calculated. Differences between subgroups were calculated using the X^2^ test or Fisher exact test. A *p*-value of 0.05 was considered significant. For performing a CT scan or operation, subgroup analyses were performed based on the performance of pelvic and acetabular surgery in the hospital and the years of experience. ‘Explanatory comments’ were divided into the CT versus no-CT and operation versus no-operation groups, if possible. If not, comments were not included in the statistical analysis. Missing data were not replaced with substituted values.

## 3. Results

### 3.1. Respondents

Of the possible 499 respondents, 189 respondents from different hospitals all over the country filled out the questionnaire. Twenty respondents were excluded from further analysis because of incomplete clinical vignettes. This resulted in 169 surveys being included for further analysis.

**Figure 1:**
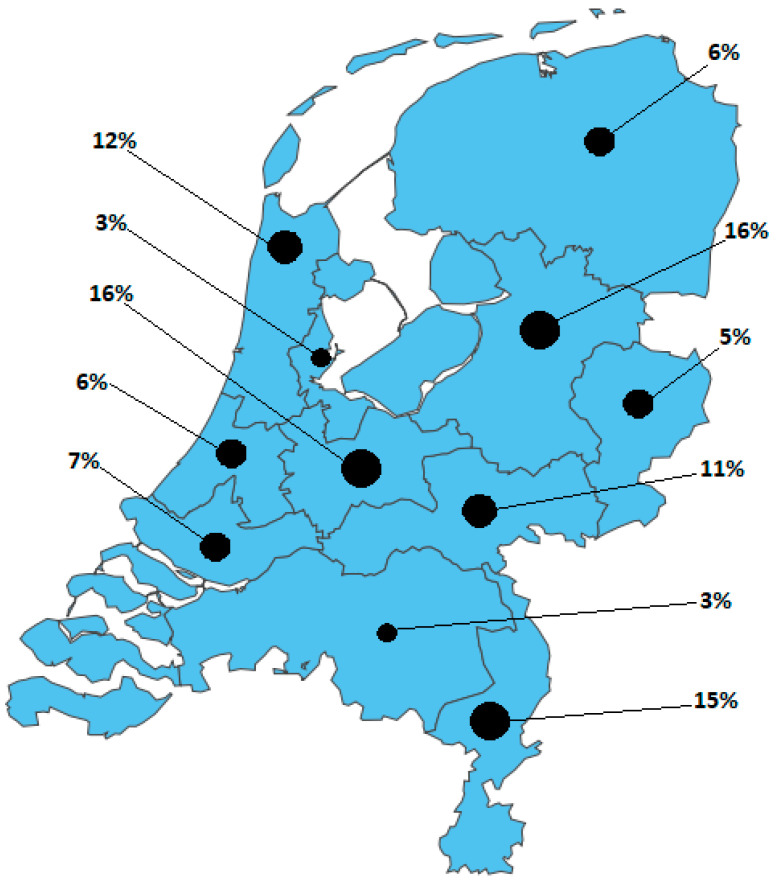
Distribution of survey respondents over the different trauma regions in the Netherlands.

Overall, 136 (37%) of the 370 registered trauma surgeons and 53 (41%) of the 129 registered orthopedic surgeons who treat fractures in the Netherlands completed the survey. Figure 1 shows the dispersion of respondents from various regions in the Netherlands. Of the respondents, 72% are trauma surgeons, and 28% are orthopedic surgeons. Most respondents had 10–20 years of work experience (34%). Of the respondents, 29% work in a level 1 Trauma Center, 54% in a level 2 Trauma Center, and 17% in a level 3 Trauma Center. A total of 47% of the respondents work in a hospital performing P&A fracture surgery, and 27% of the respondents perform P&A fracture surgery themselves.

Two-thirds of the respondents either do not have a local treatment protocol for older adult patients with a pelvic fracture or are not aware if there is such a treatment protocol in their hospital.

When asked if the respondents who do not work in a hospital performing P&A fracture surgery ever refer patients to a specialized pelvic center for surgical fixation, the majority (77%) responded that they refer patients 1–5 times a year.

### 3.2. Imaging

We presented the respondents with two patients who showed a unilateral superior and inferior ramus fracture on a plain radiograph. In patient A, a 75-year-old woman with ASA class 2, 32% of the respondents would always perform a CT scan, 55% if the patient was painful during manual sacral pressure or mobilization, and 11% would not perform a CT scan at all. By 2% of the respondents, an answer was given that could not be fitted into one of these categories.

**Figure 2:**
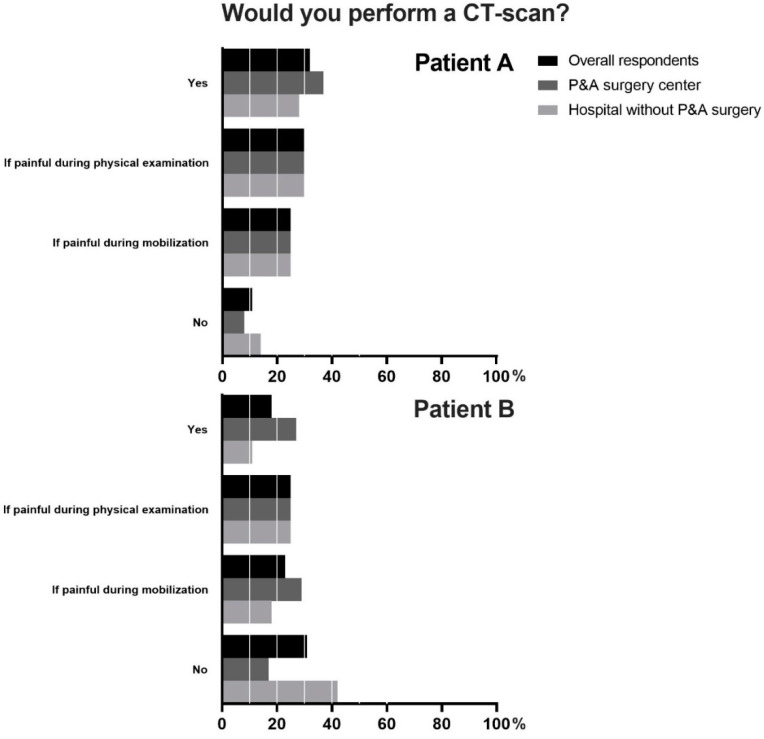
Performance of a CT scan in a 75-year-old woman with ASA 2 (patient **A**) and a 77-year-old woman with ASA 4 (patient **B**) with a ramus superior/inferior fracture on plain radiographs.

In contrast, in patient B, a 77-year-old woman with ASA 4, 18% of the respondents would always perform a CT scan, 48% if the patient was painful during manual sacral pressure or mobilization, and 31% of the respondents would not perform a CT scan at all. The reason given for not performing a CT scan was that the respondents felt it would not have treatment consequences for these patients. For details, see Figure 2.

When comparing the data of the respondents who do and do not work in a P&A surgery hospital, we see that 8% vs. 13% of the respondents would not advise a CT scan for patient A, respectively. However, there is a significant difference when comparing the data for patient B: 17% of the respondents working in a P&A hospital would refrain from making a CT scan vs. 43% of the respondents who do not (*p* = 0.002). We see that 90% of the respondents working in a P&A hospital who advise performing a CT scan on patient A would also advise performing a CT scan on patient B. Of the respondents not working in a P&A hospital, 66% of the respondents who advise for a CT scan in patient A would also advise for a CT scan in patient B.

When comparing the data of the respondents with 0–10 years of experience as an (orthopedic) trauma surgeon to the data of the respondents with >10 years of experience, we see little difference. Of the respondents with 0–10 years of experience, 79% of the respondents would advise a CT scan in both patients A and B, and 76% of the respondents with >10 years of experience would advise a CT scan in both patients A and B.

### 3.3. Treatment and Referral

The respondents were presented with three vignettes of a patient with an FFP type 3c on a pelvic radiograph and CT scan. In patient C, a 75-year-old woman with ASA class 2, 21% of the respondents would advise against surgical fixation because this would have little impact on the patient’s outcome and 1% because the surgery would be too invasive. The majority would advise surgical fixation if the patient was either painful during manual pelvic pressure (42%) or if the pain did not decrease at 6 weeks of follow-up (24%). Ten percent of the respondents would contact a P&A referral hospital to help with their decision.

When presented with patient D, a 69-year-old woman with ASA 3 and COPD Gold 3, and patient E, an 81-year-old woman with ASA 3, even more respondents deem the patient not eligible for surgical fixation. In patient D, 24% of the respondents feel surgical fixation has little impact on the patient’s outcome, and 10% feel fixation would be too invasive for the patient. In patient E, 24% and 17% of the respondents would refrain from surgical fixation for the same reasons.

**Figure 3:**
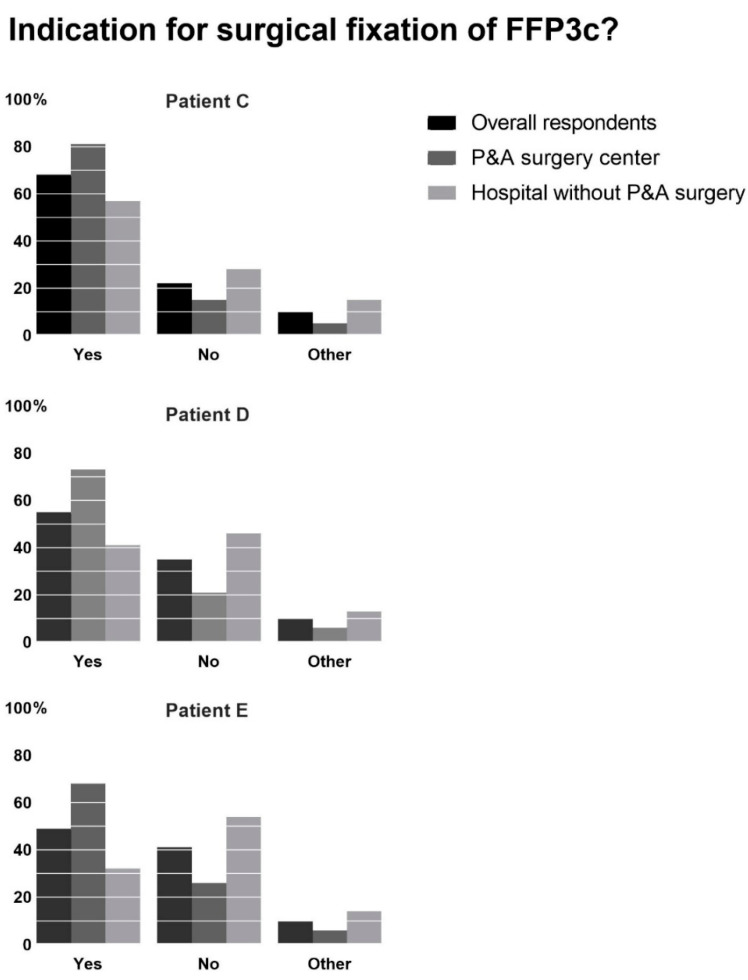
Surgical fixation of a 75-year-old woman with ASA 2 (patient **C**), a 69-year-old woman with ASA 3 (patient **D**), and an 81-year-old woman with ASA 3 (patient **E**) with an FFP3c fracture on CT scan. Respondents who answered they would want to consult a specialized pelvic center before making a decision or gave different answers than yes or no were grouped as ‘other.’

When we compare the data of the respondents who do and do not work in a hospital with P&A fracture surgery, we see a difference in physician practice (see Figure 3). In patient C, 15% of the respondents who work in a P&A referral hospital would not consider surgical fixation compared to 28% of the respondents who do not work in a P&A referral hospital (*p* = 0.010). When presented with the same fracture pattern in patient E, who is older, has more comorbidities, and has more pain and problems mobilizing compared to patient C, we again see a significant difference. Of the respondents who work in a P&A referral hospital, 26% would refrain from surgical fixation, compared to 54% of the respondents who do not work in a P&A referral hospital (*p* < 0.001).

### 3.4. Aftercare

After non-operative treatment, 90% of the respondents would advise immediate weight bearing as tolerated by the patient. In contrast, immediate full weight bearing after operative fixation is advised by only 42% of the respondents, while up to 39% would postpone full weight bearing for at least four weeks. Eight percent of the respondents would not plan a follow-up appointment if an older adult patient with a superior/inferior ramus fracture was treated non-operatively.

## 4. Discussion

This survey assessed the physician practice variation in orthopedic and trauma surgeons in the Netherlands regarding the imaging and treatment of pelvic fragility fractures. We show a wide clinical variety with significant differences in the provided diagnostic strategy and treatment. Furthermore, it seems to influence whether a respondent is working at a hospital performing P&A surgery.

This survey showed that 32% would always perform a CT scan, 30% would do so if there is pain during physical examination, 25% if there is pain during mobilization, and 11% would not advise performing a CT scan in patient A. In patient B, 18% would always perform a CT scan, 25% would do so if there is pain during physical examination, 23% if there is pain during mobilization, and 31% would not advise performing a CT scan. In this study, the most important indicator to perform an additional CT scan, if not performed immediately following conventional radiographs, is tenderness or pain on palpation of the sacrum and sacroiliac joint. The significance of clinical examination specifically for the detection of posterior pelvic fractures has not been extensively researched. However, a recent study showed promising results, describing that clinical examination predicts posterior fractures in 83% of cases [15]. So, there is some rationale for performing a CT scan under certain circumstances instead of routine CT. In addition, 11% (ASA 2 patients) and 31% (ASA 4 patients) of the respondents answered not to perform a CT scan, mostly due to the idea that it would not have treatment consequences. Posterior pelvic fractures are frequently missed on X-ray imaging, so without advanced imaging, the full scope of the injury and the indication for surgical fixation remain unknown [7,8,16]. The treatment possibilities have recently shifted to minimally invasive percutaneous techniques, and there has even been a small case series describing the possibility of percutaneous fixation under spinal anesthesia for pelvic fragility fractures [17]. There might be a knowledge gap for physicians not exposed to pelvic and acetabular surgery who think that in older adult patients, surgical fixation still entails invasive open procedures.

While the use of additional CT scans seems to be a topic of debate in clinical practice, in recent literature, the necessity for advanced imaging to thoroughly analyze the full fracture pattern seems to be almost undisputed. Most studies focusing on the treatment of pelvic fragility fractures perform CT imaging if a fracture is seen on an X-ray or there is a clinical suspicion of a sacral fracture [13,18,19,20], and some studies even advise for MRI imaging in a select group of patients [21,22,23]. This disparity IN clinical practice might be due to publication bias or can be explained by practice pattern variation between different countries.

In the three cases of a patient with an FFP type 3c confirmed on a CT scan, a notable difference is seen. In patient C, 81% and in patient E, 68% of the respondents working in a P&A hospital would advise surgical fixation. In contrast, 57% and 32% of the respondents not working in a P&A hospital would advise surgical fixation, respectively. The main goal of treatment for pelvic fractures, whether conservative or surgical, is to sustain mobility and consequently prevent the negative effects of prolonged immobility [24,25]. According to the most recent studies, fracture type, mobility level, and pain should all influence the decision to proceed with surgical treatment [26,27,28,29,30,31]. This survey shows that after 2 weeks of reduced mobility and pain, surgical stabilization is not recommended by many treating physicians, especially in frail patients. A recent literature review encompassing the surgical treatment of all types of FFPs found consistent improvement in pain and mobility after percutaneous surgical fixation and found that operative complications were uncommon [14]. Preserving mobility is especially important in older adult patients, as disuse of the lower limbs for as little as 4 days can cause long-term reduced mechanical muscle function [32]. The same disparity is seen regarding the decision to perform a CT scan or to refer a patient to a specialized pelvic center for surgical fixation. The respondents’ exposure to pelvic and acetabular surgery seems to be a factor in this decision.

Current treatment strategies for patients suspected of FFPs currently seem highly dependent on where or to whom the patient is presented. Because of the overall more complex nature of operation techniques, it is universally accepted that surgical treatment of pelvic fractures is performed in P&A-specialized referral trauma centers. Generally, high exposure (centralization) leads to more experience and fewer complications. However, this survey shows it may also lead to an increase in unfamiliarity with this type of fracture and its treatment options outside of centralized care. A possible solution may be the implementation of regional and/or national treatment guidelines that summarize effective treatments and recommended advanced imaging and include a referral strategy to specialized pelvic centers.

### Strengths and Limitations

The strength of this survey is the unique insight it offers into the variation in physician practice regarding the diagnosis and treatment of FFPs. Identifying practice pattern variation is the first step towards standardized care and highlights the topics that should be addressed in future guidelines. As far as the authors are aware, no other studies focusing on physician practice variation in the diagnostic and treatment strategies for older adults with pelvic fractures have been performed. Since the survey was completed by respondents from different trauma centers all over the country and included high numbers of both pelvic surgeons and those who do not operate pelvic fractures, the results of this survey can be generalized to a larger population. However, the proportion of respondents who perform P&A surgery themselves is higher than the expected national proportion: 27% of the respondents in the survey vs. 11% nationwide, which might be an indicator of sampling bias.

The response rates in doctor surveys in the field of surgery vary, and there is no consensus on the benchmark for a ‘good’ or ‘excellent’ response rate. While in-person surveys score relatively high, web-based doctor surveys, on average, have a 46 ± 25% response rate [33]. Our response rate of 38% is on par with these numbers.

This survey study has several limitations. In clinical practice, physicians assess a patient by combining both patient-specific characteristics and external factors when deciding on diagnostics and treatment strategies. In this survey, data and conclusions were derived from a subset of only five clinical vignettes, which might be oversimplified. However, this type of survey offers the opportunity to isolate physicians’ decision-making and to control case-mix variation to assess practice variation.

The design of a survey study is inherent in its limitations and biases. Because of the anonymous nature of the survey, it remains unknown how the non-response/response bias affects our results, which could potentially impact the generalizability of the survey results. However, the investigators believe anonymity is an important measure to minimize social desirability bias, so respondents provide answers that reflect their true opinions or behaviors instead of answers that they believe are socially expected. To address possible experience and specialization bias, we performed a subgroup analysis based on years of experience and whether pelvic and acetabular surgery is performed in the hospital the respondent is working at. Years of experience did not impact the approach of the respondents towards diagnosis and treatment, but familiarity with the injury (e.g., pelvic and acetabular surgery performed in the hospital the respondent is working at) was a confounder.

## 5. Conclusions

There is physician practice variation regarding the diagnostic and treatment strategies of FFPs. Respondents rarely perform an additional CT scan in older adult patients with a higher ASA classification because of the belief that operative pelvic ring fixation is deemed too invasive for the patient or has little impact on the outcome. Surgical stabilization is considered too invasive for the more frail patients by many respondents. The type of hospital the patient is presented to seems to impact the imaging and treatment strategy. Although more consensus on the optimal imaging and treatment strategy for patients with FFPs is needed, an international or national treatment protocol should be implemented to ensure a more uniform approach.

## Figures and Tables

**Figure A1:**
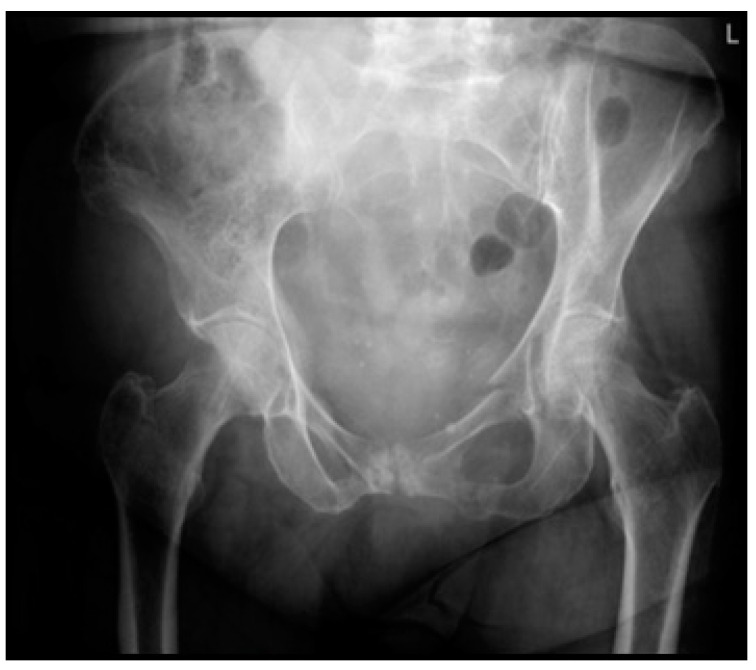
Plain Radiograph Referred to in Question 12 and 13 in the Survey Questionnaire.

**Figure A2:**
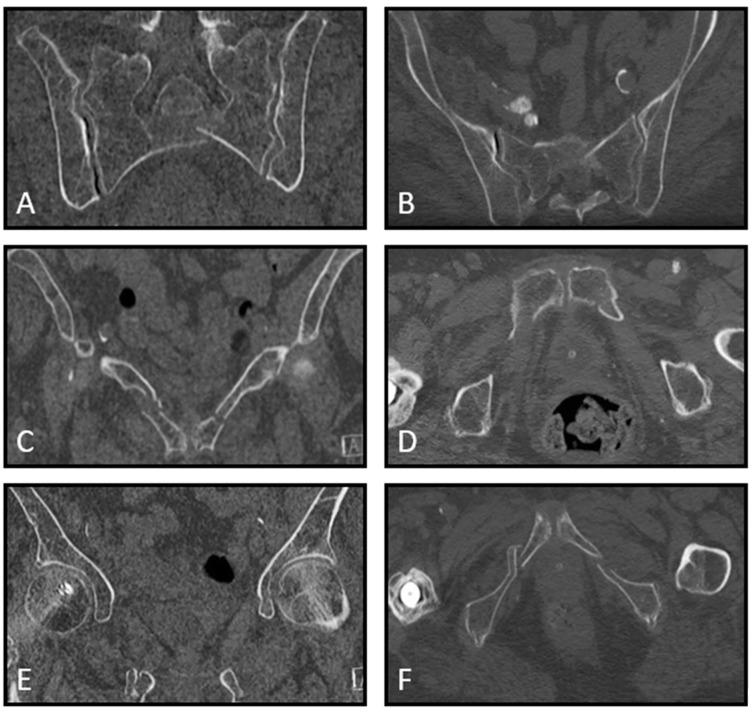
(**A**–**F**) Images of CT-Scan Referred to in Question 14–16 in the Survey Questionnaire.

## Data Availability

The data presented in this study are available on request from the corresponding author. The data are not publicly available due to the data containing information that could compromise the privacy of the participants of the survey.

## Appendix A

**Table A1:** Details of the Questionnaire.

Questions	Answer Options
1. What are you?	A. Trauma surgeon B. Trauma surgery resident C. Orthopedic surgeon D. Orthopedic surgery resident
2. For how long have you worked as a trauma or an orthopedic surgeon?	A. 0–5 years B. 6–10 years C. 11–20 years D. >20 years E. In residency
3. In which trauma care network region do you work?	A. Eastern Regional Emergency Healthcare Network B. Acute Care Network North-West C. Network Emergency Care Brabant D. Network Acute Care West E. Network Emergency Care Euregio F. Trauma Center Southwest Netherlands G. Network Acute Care Limburg H. Network Emergency Care Zwolle I. Trauma Network Middle Netherlands region J. AcuteCareNet AMC K. Acute Care Network North Netherlands
4. Which Trauma Center Level meets your hospital?	A. Level 1 (highest) B. Level 2 C. Level 3 (lowest)
5. Are pelvic and acetabular fracture operations performed in your hospital?	A. Yes, 1–20 operations per year B. Yes, 21–40 operations per year C. Yes, 41–60 operations per year D. Yeas, >60 operations per year E. No
6. Do you perform pelvic and acetabular fracture operations?	A. Yes, 1–10 operations per year B. Yes, 11–20 operations per year C. Yes, 21–40 operations per year D. Yes, >40 operations per year E. No
7. How many elderly patients (≥65 years) with a superior/inferior ramus fracture on plain radiographic imaging do you treat in your hospital (both operatively and conservatively)?	A. 1–25 patients per year B. 26–50 patients per year C. 51–75 patients per year D. 76–100 patients per year E. >100 patients per year
8. How often do you refer elderly patients with pelvic fractures after low-energy trauma to a specialized pelvic center?	A. 1–5 times per year B. 6–10 times per year C. >10 times per year D. Not applicable
9. Who generally assesses an elderly patient with a superior/inferior ramus fracture in your hospital’s Emergency Department (ED)?	A. Surgeon/orthopedic surgeon B. Surgical/orthopedic residents (not in training) C. Surgical/orthopedic residents (in training) D. Emergency physician E. Otherwise, namely:
10. In case an elderly patient with a superior/inferior ramus fracture needs to be admitted to the hospital, where will this patient be admitted?	A. Surgical ward/orthopedic ward B. Geriatric ward C. Nursing home D. ‘Primary care’ nursing home E. Otherwise, namely:
11. Is there a treatment protocol for elderly patients with a superior/inferior ramus fracture in your hospital?	A. Yes, named: B. No C. I am not aware if there is such a treatment protocol
12. Mrs. A. is a 75-year-old, independently living ASA 2 patient. She tripped and fell at home. Plain radiographic imaging showed a superior/inferior ramus fracture on the left side. Would you, based on this information, perform a CT scan in the hospital you work at?	A. No, a CT scan has no treatment consequences B. Yes, always C. Yes, if the patient has pain on palpation of the sacrum D. Yes, if the patient is very painful during mobilization E. Otherwise, namely:
13. Mrs. B. is a 77-year-old ASA 4 patient living in sheltered housing. She tripped and fell at home. Plain radiographic imaging showed a superior/inferior ramus fracture on the left side. Would you, based on this information, perform a CT scan in the hospital you work in?	A. No, a CT scan has no treatment consequences B. Yes, always C. Yes, if the patient has pain on palpation of the sacrum D. Yes, if the patient is very painful during mobilization E. Otherwise, namely:
14. Mrs. C. is a 75-year-old, independently living ASA 2 patient. She fell on the street, and plain radiographic imaging showed a superior/inferior ramus fracture on the right side. She is discharged from the ER with oral painkillers. The patient comes back to your outpatient clinic after 14 days. She mobilizes with difficulty, sits in a wheelchair, walks with a four-wheel walker indoors, and uses Paracetamol and Diclofenac. A CT scan shows an LC1/FFP3c fracture. Do you think, based on this information, the patient is eligible for surgical fixation?	A. No, I do not think this is indicated and would have little impact on this patient’s outcome B. No, I think operative pelvic fixation would be too invasive for this patient C. Yes, if the patient has evident pelvic pain during a physical exam D. Yes, only if the pain did not decrease at 6 weeks follow-up E. Otherwise, namely:
15. Mrs. D. is a 69-year-old, independently living ASA 3 patient with COPD Gold 3. She fell on the street, and plain radiographic imaging showed a superior/inferior ramus fracture on the right side. She is discharged from the ER with oral painkillers. The patient comes back to your outpatient clinic after 14 days. She mobilizes with difficulty, sits in a wheelchair, walks with a four-wheel walker indoors, and uses Paracetamol and Diclofenac. A CT scan shows an LC1/FFP3c fracture. Do you think, based on this information, the patient is eligible for surgical fixation?	A. No, I do not think this is indicated and would have little impact on this patient’s outcome B. No, I think operative pelvic fixation would be too invasive for this patient C. Yes, if the patient has evident pelvic pain during a physical exam D. Yes, only if the pain did not decrease at 6 weeks follow-up E. Otherwise, namely:
16. Mrs. E is an 81-year-old ASA 3 patient. She fell on the street, and plain radiographic imaging showed a superior/inferior ramus fracture on the right side. She is admitted to the nursing ward and has difficulty turning in bed. Transfer from bed to toilet chair is possible. She uses Paracetamol, Diclofenac and Oxynorm. A CT scan shows an LC1/FFP3c fracture. Do you think, based on this information, the patient is eligible for surgical fixation?	A. No, I do not think this is indicated and would have little impact on this patient’s outcome B. No, I think operative pelvic fixation would be too invasive for this patient C. Yes, if the patient has evident pelvic pain during a physical exam D. Yes, only if the pain did not decrease at 6 weeks follow-up E. Otherwise, namely:
17. If plain radiographic imaging shows a superior/inferior ramus fracture and the patient is treated conservatively, when would your first follow-up moment be?	A. No follow-up B. Within 1 week C. Within 2 weeks D. Within 3 to 6 weeks E. After >6 weeks
18. If plain radiographic imaging shows a superior/inferior ramus fracture and the patient is treated conservatively, when would you advise the patient to use full weight bearing?	A. Immediately, based on the patient’s pain B. After 2 to 4 weeks C. After 4 to 6 weeks D. After 6 to 8 weeks E. Otherwise, namely:
19. If a patient underwent operative fixation related to a right-sided sacrum and superior/inferior ramus fracture, when would you advise the patient to use full weight bearing?	A. Immediately, based on the patient’s pain B. 2 to 4 weeks minimal weight bearing, afterward full weight bearing C. 4–6 weeks minimal weight bearing, then full weight bearing D. 6 to 8 weeks minimal weight bearing, then full weight bearing E. Otherwise, namely:
